# “Everyone's Responsibility and No‐One's Responsibility”: A Thematic Analysis of a Roundtable on the Complex Problem of Secondary Stroke Prevention in Australia

**DOI:** 10.1111/hex.70755

**Published:** 2026-07-06

**Authors:** Seamus Barker, Dominique Cadilhac, Jan Cameron, Marie‐Louise Bird, Liam Johnson, Coralie English, Kyra Hamilton, Robyn A. Clark, Timothy Kleinig, Stephanie Smith, Beth Crane, Brian Beh, Kim Beesley, Jennifer Muller, Ramesh Sahathevan, Helen Dewey, Carlos Garcia‐Esperon, Mark Nelson, Seana Gall

**Affiliations:** ^1^ Menzies Institute for Medical Research University of Tasmania Hobart Australia; ^2^ Stroke and Ageing Research, Department of Medicine, School of Clinical Sciences at Monash Health Monash University Clayton Melbourne Australia; ^3^ Stroke Theme, Florey Institute of Neuroscience and Mental Health University of Melbourne Melbourne Australia; ^4^ School of Health Science, College of Health and Medicine University of Tasmania Hobart Australia; ^5^ School of Behavioural and Health Sciences Australian Catholic University Melbourne Australia; ^6^ School of Health Sciences, College of Health, Medicine and Wellbeing University of Newcastle Callaghan Australia; ^7^ Heart and Stroke Program Hunter Medical Research Institute New Lambton Heights Australia; ^8^ School of Applied Psychology Griffith University Brisbane Australia; ^9^ College of Nursing and Health Sciences Flinders University Adelaide Australia; ^10^ Adelaide SA, Medical School, Faculty of Health and Medical Sciences University of Adelaide Adelaide Australia; ^11^ School of Psychological Sciences, College of Health and Medicine University of Tasmania – Inveresk Campus Launceston Australia; ^12^ Stroke Foundation Melbourne Australia; ^13^ Independent Lived Experience Expert Sydney Australia; ^14^ Lived Experience Council, Stroke Foundation Melbourne Australia; ^15^ School of Medicine Deakin University Melbourne Australia; ^16^ Eastern Health Clinical School, Faculty of Medicine, Nursing and Health Sciences Monash University Melbourne Australia; ^17^ School of Medicine and Public Health University of Newcastle Newcastle Australia; ^18^ School of Clinical Sciences Monash University Melbourne Australia

**Keywords:** prevention, roundtable, secondary prevention, secondary stroke, stroke, systems thinking, thematic analysis

## Abstract

**Background:**

Around one in four people with stroke will have a recurrent stroke. The risk can be reduced through medication and lifestyle changes, but many have poor risk factor control post‐stroke. There is no nationally recognised model of care for stroke secondary prevention, and inadequate secondary prevention may be a complex problem that cannot be effectively dealt with by addressing various contributing factors independently. We sought to understand implementation and research priorities to address gaps in stroke secondary prevention in Australia.

**Methods:**

An online roundtable and four follow‐up small group discussions were conducted between March and April 2023. Participants were people with lived experience of stroke, researchers, health professionals and representatives from stroke advocacy organisations and the Australian federal government. Participants were given pre‐discussion questions on stroke secondary prevention priorities, barriers and enablers. Moderated discussion invited reflection on system‐level connections between priorities and shared understanding of diverse perspectives, rather than consensus‐building. Discussions were recorded, transcribed and analysed following Braun and Clarke's thematic analysis methodology.

**Results:**

In the roundtable and small‐group discussions, 25 people participated (68% women). Most priorities related to the inductive theme, ‘models of care’, including programmes, interventions and health system settings. Perceived barriers and enablers related to the following inductive themes: ‘coordination’, ‘resourcing’, and the ‘research translation pipeline’. Identified priorities were heterogeneous and extensive. Tensions existed between some priorities, but others were shared by participants.

**Conclusion:**

Our roundtable identified a range of priorities for stroke secondary prevention, coming from diverse perspectives. Stroke secondary prevention is a complex problem with interacting system elements that make improvement challenging. A model of care for stroke secondary prevention in Australia is needed that accounts for this complexity while building upon shared priorities.

**Patient or Public Contribution:**

This project embedded people with lived experience of stroke. This included three people with lived experience of stroke, or of being a carer for a person with stroke, who took part in the initial roundtable and provided valuable contributions in discussions of implementation and research priorities for secondary stroke prevention. These participants also helped to prepare this manuscript, providing helpful feedback to ensure that priorities, barriers and enablers for secondary stroke prevention from a lived experience perspective were centred.

## 1 Introduction

Stroke is the second most common cause of death and the third leading cause of disability in the world [1], affecting all ages, ethnicities and socio‐economic groups [2, 3]. While stroke is a highly preventable disease, the economic costs and years lived with stroke‐related disability continue to increase due to population ageing and growth. There is a trend towards increasing prevalence of some stroke risk factors and greater survival among people who have a stroke [4].

Up to 25% of people who have a first stroke will go on to experience a recurrent event within the next 5 years [5]. Each year, 46,000 strokes occur in Australia [6], 30% (13,800) of which are recurrent strokes [7]. After a first stroke or transient ischaemic attack (TIA), the risk of another stroke within the next 12 months is elevated by eight‐fold, and can remain elevated by six‐fold for up to 10 years [8]. Many recurrent strokes could be prevented through effective modification of known risk factors.

Unfortunately, risk factor control can be a challenge for people with stroke. Few people quit smoking [9], many have poorly managed hypertension [10], most are physically inactive [11], and there is an under‐prescription of anti‐hypertensive, anti‐lipidemic and anti‐platelet medication [12]. Data from the Australian Stroke Care Registry reveal that, in 2021, 31% of people who had a stroke in Australia did not receive a discharge care plan from the hospital, which includes secondary prevention guidance for the patient and general practitioner (GP) [13]. Most people with stroke receive secondary prevention medications at discharge from hospital, but up to a third cease taking them within 1 year [14]. Of people with stroke who have been followed at 3–6 months after discharge, 71% reported unmet needs in secondary prevention [15]. These data suggest that secondary prevention of stroke in Australia is suboptimal.

Currently, there is no standardised model of care for secondary prevention of stroke. In contrast, secondary prevention of heart disease has an established model of care—cardiac rehabilitation—although challenges have been identified in its implementation [16]. In response to these challenges, the Australian Cardiovascular Alliance (ACvA) conducted a roundtable in 2019, which established Australian implementation and research priorities relating to cardiac secondary prevention and rehabilitation [17]. In 2023, ACvA, in partnership with the Cardiac Society of Australian and New Zealand and the National Heart Foundation of Australia, conducted another roundtable, this time focusing on implementation and policy priorities for Australia regarding cardiovascular disease (CVD) [18]. These roundtables included diverse arrays of interest‐holders and used a methodology to build consensus, resulting in a roadmap to improve secondary prevention for heart disease, including shortlists of priorities, goals and actions.

To date, there has not been a similar consultation about secondary prevention for people who have had a stroke. Evidence exists for interventions that can modify risk factors after stroke [19]. However, challenges exist in implementing these evidence‐based practices in real‐world settings. This could be due to the complexity and variability of the target groups (i.e., people with stroke and different health professions involved in care for people with stroke); contexts in which these interventions are delivered (e.g., acute, rehabilitation and primary care); and interventions themselves (e.g., multimodal behaviour change programmes). This complexity is likely part of the reason why there is no standardised model of care for secondary prevention of stroke in Australia. Systems thinking may assist in understanding and addressing complex issues like secondary prevention of stroke [20]. Systems thinking is not a methodology but rather an epistemology that understands synergistic interactions between elements of complex systems to result in emergent features, making planned change difficult [21]. Consultation with interest‐holders and people with lived experience of a condition can be used to explore an issue from a systems thinking perspective [22].

Since there is no standardised model of care for recurrent stroke prevention—and given the apparent complexity of the challenge—our objective was not consensus‐building, resulting in a shortlist of priorities. Rather, we wanted to identify and facilitate a shared understanding of a wide range of implementation and research priorities for recurrent stroke prevention in Australia. Our goal was to include a diverse array of interest‐holders, potentially with heterogeneous priorities focused on different contexts. From a systems thinking perspective, we aimed to identify perceived barriers to, and enablers of, the realisation of these priorities and potential synergies, antagonisms, and/or dependencies between them. Through this approach, we aimed to better understand how to improve stroke secondary prevention in Australia.

To meet these aims, we conducted an online roundtable and follow‐up discussions involving representatives of a diverse range of interest‐holder groups: researchers, people with lived experience of stroke, carers, neurologists, GPs, nurses, allied health clinicians and representatives from the Stroke Foundation and the Australian Government.

## 2 Methods

**Figure 1:**
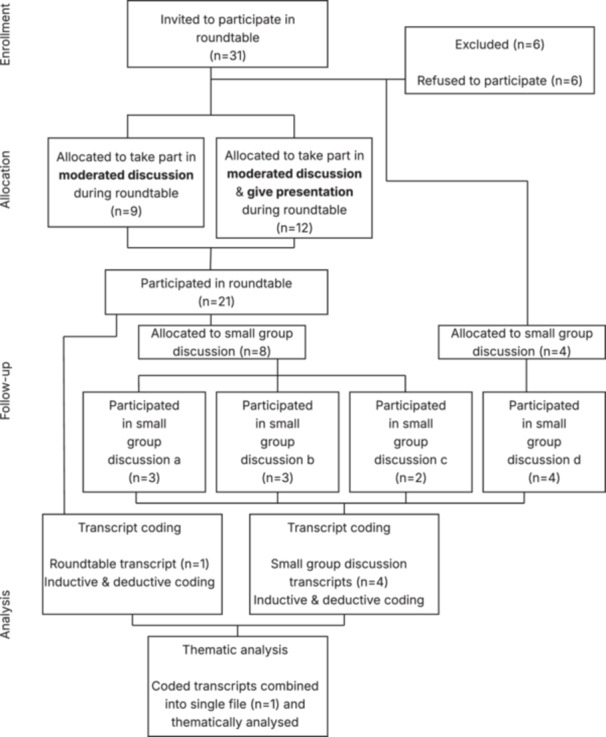
Participant flow.

The study was approved by the Tasmanian Health and Medical Human Research Ethics Committee (H0028678). A systems thinking framework informed the design. The roundtable structure, including participation and analysis process, is shown in Figure 1. At the outset, the research team identified eight broad topics reflecting different parts of the larger system within which secondary stroke prevention occurs—‘community’, ‘health systems’, ‘eHealth’ (digital health), ‘mHealth’ (mobile phone‐based health, a subset of eHealth or digital health), ‘behaviour change’, ‘primary care’, ‘continuity of care’ and ‘health policy’. Potential participants with expertise in these topics, or membership in an aforementioned interest‐holder group, were identified through purposive convenience sampling, drawing on our networks of stroke clinicians and researchers, with snowballing to identify potential participants from the Australian Government and general practice. Potential participants were approached by email by the first author. A sub‐group was invited to give presentations (individually or in a small group) during the roundtable, based on their status as thought leaders or experts (Table 2). Participation was to involve a 4‐h online roundtable with eight 15‐min presentations (details below), moderated discussion (details below), session recording and thematic analysis of de‐identified transcripts. After the roundtable, several small‐group discussions were held to either elaborate on points raised in the roundtable or involve those who could not attend the roundtable. All participants received a Participant Information Statement and provided written informed consent.

Before attending the roundtable, participants were provided with a brief (Supplement 1) informed by systems thinking. Presenters were asked to outline priorities for implementation and/or research in secondary stroke prevention in Australia, considering:

- Whether these priorities are currently implemented or studied.
- Barriers to the achievement of.
- Factors that could enable the achievement of.

For the moderated discussion, all participants were asked to reflect on:

- What is needed for evidence‐based interventions for secondary stroke prevention to be implemented and scaled.
- Connections (synergies, antagonisms, dependencies) between priorities.
- What a ‘blue sky’ solution might look like.
- Concrete, realistic changes in policy, funding, or health system organisation that could enable achievement.

Small‐group participants who had not joined the roundtable received the same brief. They did not prepare presentations but outlined priorities, considering the same points.

At the roundtable's start, the last author introduced themselves and explained their reasons for undertaking the research. After each presentation, a moderated discussion took place, facilitated by S.B. (male), at that time a PhD Candidate with experience facilitating focus groups. He was chosen because he was not a cardiovascular researcher and had no biases regarding secondary stroke prevention, though his interest in systems thinking influenced his moderation. Before the discussion, he reiterated points from the brief (Supplement 1). Moderation involved managing questions, prompting reflections and inviting responses aligned with the brief. After all presentations, participants were invited to consider potential connections (synergies, antagonisms, dependencies) between identified priorities.

The roundtable and small‐group discussions were recorded via Zoom and transcribed in de‐identified form S.G. took field notes during the roundtable; S.B. did so during small‐group discussions.

**Figure 2:**
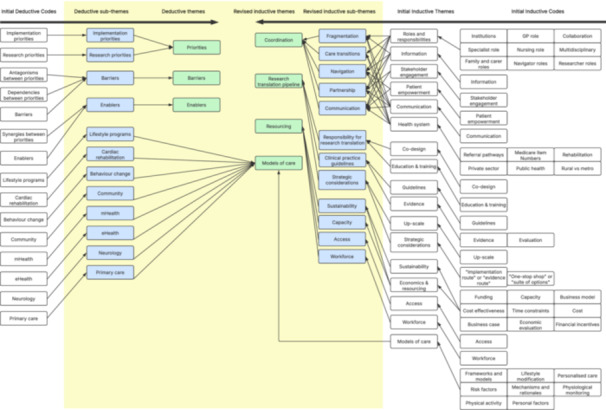
Coding tree.

A thematic analysis was undertaken using the Consolidated criteria for reporting qualitative studies (Supplement 2). N‐Vivo was used for the analysis. Initial coding was completed by S.B., an experienced qualitative researcher (by then with a doctorate in sociology). Deductive codes were derived from the brief (Supplement 1), based on study aims and prioritised topics. Transcripts were then read, inductive codes derived and instances of deductive codes documented. A coding tree was developed (Figure 2). Codes were cross‐checked by S.G., S.S. and B.C. The five transcripts (i.e., from the one roundtable and four small group discussions) were combined into a single file. S.B. completed another round of coding using both inductive and deductive codes. Codes were consolidated into higher‐level themes by S.B. S.G., S.S. and B.C. independently coded and generated themes, then discussed findings with S.B. until agreement was reached. Final codes and themes were incorporated into the coding tree (Figure 2).

Consistent with our systems thinking approach, instances of simultaneous coding were identified, where text fragments were coded to multiple themes [23]. We produced brief paraphrase summaries of these instances, and a table recording the different themes to which each summary was coded (Supplement 3). We split this large table into smaller tables, each organised by a different inductive theme, to demonstrate interrelationships between themes (Tables 4, 5, 6, 7).

Transcripts were not returned to participants for comment. Instead, participants provided feedback on drafts of the thematic analysis manuscript, which was incorporated into subsequent drafts. Data saturation was not sought, though follow‐up small‐group discussions ceased once sufficient ‘information power’ was achieved to meet the study's aims [24].

## 3 Results

The participation flow chart is shown in (Figure 1). We invited 31 people to participate, with 14 potential participants previously engaged with the research team's work in stroke secondary prevention. The roundtable took place in March 2023 as an online event using Zoom, lasting 4 h. There were 21 participants with diverse demographic characteristics and representative of a wide range of interest‐holder groups (Table 1), and two observing non‐participants affiliated with the research team. The details of the 12 thought‐leader or expert participants who gave presentations during the roundtable are shown in Table 2. After the roundtable, four small‐group discussions were held with three to elaborate on points raised in the roundtable and one with those who could not attend the roundtable.

**Table 1:** Participant characteristics.

			Roundtable participants 21	Small‐group participants (who did not participate in roundtable) 4	Combined 25
Demographics	Gender	Female	16 (76)	1 (25)	17 (68)
	Location	New South Wales	5 (24)	1 (25)	6 (24)
		Victoria	4 (19)	2 (50)	6 (24)
		Queensland	4 (19)	0	4 (16)
		Western Australia	2 (10)	0	2 (8)
		South Australia	1 (5)	1 (25)	2 (8)
		Tasmania	3 (14)	0	3 (12)
		Australian Capital Territory	2 (10)	0	2 (8)
		Northern Territory	0	0	0
Interest‐holder group representation (*N.B. some participants were classified in two stakeholder categories, so interest‐holder group representation totals ≥ participant totals*)		Lived experience	3 (14)	0	3 (12)
		Researcher	14 (67)	4 (100)	18 (72)
		Neurologist	0	4 (100)	4 (16)
		General practitioner	2 (10)	0	2 (8)
		Nurse	4 (19)	0	4 (16)
		Allied health	6 (29)	0	6 (24)
		Australian Government	3 (14)	0	3 (12)
		Stroke Foundation	4 (19)	0	4 (16)

*Note:* All values *n* (%).

**Table 2:** Individual presentation topics and interest‐holders.

Individual presentation topics	Backgrounds of topic experts
Community	Researcher/physiotherapist
Health systems	Researcher/exercise scientist
mHealth	Researcher/nurse
eHealth	Researcher/physiotherapist
Behaviour change	Researchers/psychologist/nursing
Primary care	Researcher/nurse
Continuity of care	Stroke Foundation
Healthy policy	Australian Government/general practice

Fifteen minute presentations were given on the following topics by thought leaders/expert participants (Table 2). These broad topics were chosen by the research team on the basis that they reflect different parts of the larger system within which secondary stroke prevention occurs.

In March and April 2023, four small‐group discussions were held (Figure 1a–d). Each was conducted online using Zoom and lasted 1 h (Table 3).

**Table 3:** Small group discussions: Purpose and interest‐holders.

Discussion purpose	Discussion focus	Number of participants	Interest‐holder groups represented
Elaboration of points raised roundtable (participants had taken part in roundtable)	Behaviour change	3	Researcher/psychologist/nursing
	Primary care	3	Researcher/nurse/general practitioner
	System‐level barriers and enablers	2	Researcher/physiotherapist
Identify priorities from perspective of neurology (participants had not taken part in roundtable)	Implementation and/or research priorities	4	Neurologist/researcher

The deductive codes derived from the brief and the inductive codes derived from the roundtable and small‐group discussion transcripts appear in a coding tree in Figure 2 above.

### 3.1 Inductive Themes

#### 3.1.1 Models of Care

There was considerable discussion of interventions, care settings, modalities, outcomes, mechanisms of action and factors affecting secondary stroke, such as physical activity and risk factors. Much of this discussion related to implementation and research priorities for secondary stroke prevention and often focused on the topics on which presentations were given (e.g., behaviour change, mHealth, eHealth, primary care).

#### 3.1.2 Coordination

In identifying barriers to, and enablers of, the realisation of priorities for secondary stroke prevention, there was much discussion of fragmentation of the health system, coordination of care, communication (or the lack of) between individuals, services and institutions, care transitions for people with stroke, navigation of services, formal and informal partnering, the transmission of information, different roles and responsibilities in secondary stroke prevention, stakeholder engagement and patient empowerment. Discussions of these points also described synergies, antagonisms and dependencies between different implementation and/or research priorities.

#### 3.1.3 Resourcing

Many of the identified barriers to realising implementation and research priorities related to funding, workforce, capacity, access to services, sustainability of services and programmes, costs to patients, time constraints and antagonisms or dependencies between different priorities. Enablers were also identified relating to the use of economic evaluations, creating robust business models and business cases, ensuring cost‐effectiveness of programmes, utilising financial incentives to create behaviour change, and synergies between different priorities.

#### 3.1.4 Research Translation Pipeline

Both implementation and research priorities frequently related to the research translation pipeline. Many barriers to, and enablers of, the realisation of these priorities were discussed. These related to the question of responsibility for research translation, clinical practice guidelines, various strategic considerations, scaling, evidence, evaluation, the role of education and training, co‐design and how to translate evidence‐based interventions into routine care.

### 3.2 Simultaneous Coding to Multiple Themes

During coding, simultaneous coding to multiple themes—both inductive and deductive—was commonplace. We identified 102 instances of simultaneous coding and produced paraphrase summaries of these. A table including these 102 paraphrase summaries and the themes to which they were coded appears in Supplement 2. From this larger table, we developed four smaller tables, each organised by one of the inductive themes (models of care, coordination, resourcing, or research translation pipeline) and two deductive themes (barriers and enablers, or research and implementation priorities). The 102 paraphrase summaries were entered into these tables according to how they had been simultaneously coded, as described below. Due to the heterogeneity and volume of unique discussion points, we present these 102 paraphrase summaries in tables below, rather than presenting direct quotations from participants.

The inductive theme, ‘models of care’, had most instances of simultaneous coding with the deductive theme, ‘priorities’. The identified priorities relating to models of care were heterogeneous and oriented to a diverse range of contexts, including different secondary prevention programmes, modalities, health system settings and health professions focusing on research or implementation (Table 4).

**Table 4:** Simultaneous coding to themes: ‘Models of care’ and ‘priorities’.

	Research priorities	Implementation priorities
Multimodal programmes	Determining the most effective and cost‐effective programme model (e.g., supervised exercise plus education)	Access to multimodal programmes whenever the person living with stroke is ready—such programmes being available at different points in care continuum
	Determining the ‘active ingredients’ of successful multimodal interventions	
Cardiac rehabilitation	Determining whether a ‘cardiac rehabilitation’ model is appropriate for stroke secondary prevention	Equity for people with stroke having access to state‐funded secondary prevention comparable to cardiac rehabilitation for cardiac patients
Behaviour change programmes	Evaluate programmes that develop behaviour change capacities	New service models to support behaviour change
		Translate evidence‐based behaviour change programmes into usual care
Community		Personalised, culturally safe lifestyle medicine
mHealth	Producing evidence into effectiveness and cost‐effectiveness of different mHealth interventions compared with usual care	As high‐level evidence is gathered, integrating mHealth interventions synergistically with other service models as part of usual care
		Pipeline from pilot studies to fully powered trials to translation into routine care
eHealth		eHealth resources for behaviour change support should focus on implementation rather than gathering evidence of effectiveness
		Integrating eHealth with other interventions in larger rehabilitation and community service models
Neurology		After stroke, every patient should have a timely follow‐up with a neurologist with expertise in stroke for a secondary prevention plan
		National, multidisciplinary, evidence‐based, multimodal behaviour change programme for people with stroke
		Personalised secondary prevention targets
		Clinical practice guidelines that have clear, evidence‐based recommendations for direct oral anticoagulants (DOACs), antiplatelets and blood pressure, blood glucose and lipid targets
		Ensuring neurologists remain up to date with evidence‐based treatment options, including for glucose and lipid management
Primary care	Establishing evidence for novel primary care models for stroke secondary prevention	General practitioners involved in behaviour change support and overseeing secondary prevention management in the community
Overlapping	Determining how programmes can be sustainably implemented	Improving reach and uptake of secondary prevention interventions and programmes through personalised care
Contested/uncertain	Building evidence vs translation or implementation	Single national programme for stroke secondary prevention vs plurality of interventions and programmes
	The role of the researcher in implementation and translation	

The inductive theme, ‘coordination’, had most instances of simultaneous coding with the deductive themes, ‘barriers’ and ‘enablers’. Perceived barriers and enablers associated with coordination were heterogeneous, relating to fragmentation of the health system, coordination of care, communication (or the lack of), care transitions for people with stroke, navigation of services and formal and informal partnering, across a range of contexts and models of care (Table 5).

**Table 5:** Simultaneous coding to themes: ‘Coordination’, ‘barriers’ and ‘enablers’.

Barriers	Enablers
Fragmentation of health system	Embedding patient autonomy and choice (person‐centred care) within stroke secondary prevention programmes to improve partnering between patients and clinicians
Minimal coordination between specialists; specialists and general practitioners (GPs); GPs and allied health; researchers and policymakers; researchers and funders	‘Shared care’ models to improve partnering between different treating clinicians
No clear pathway for implementation of research into routine care	Communication between specialists and general practice can be improved through standardisation and automation of letters
Inadequate communication between levels and services of health system; clinicians, families and caregivers	Creating more partnerships between interest‐holders, clinicians, patients and families, including through formal multi‐disciplinary teams
Different clinical pathways after stroke/TIA for those going to rehabilitation or directly home creates complexity in coordination across levels and specialities within health care system	Cardiac rehabilitation model of care featuring menu of options can enhance reach and uptake—could be adapted for stroke
No standardised model of care for stroke secondary prevention results in lack of coordinated care across hospital and primary care, in contrast with post cardiac event.	Cardiac rehabilitation model set in primary care can enhance reach and uptake—could be adapted for stroke
Responsibility of many disciplines and parts of the health and community sectors	Model of care using existing Medicare Benefits Schedule (MBS) item numbers to coordinate multidisciplinary, multimodal primary care (like primary care‐based cardiac rehabilitation model)
Despite common risk factors for different chronic conditions, particularly cardiac conditions, health system divides by disease and body system	Programmes that develop behaviour change capabilities
Behaviour change is difficult to achieve	Peer‐led models for secondary prevention support
Low technology literacy in patients	Family and community members supporting transition to community living
Patient preference	Clinician training to form effective partnerships with families and carers
Need for oversight of physiological monitoring through mHealth and eHealth	Clinical practice guidelines requiring clinicians to form effective partnerships with families and carers
Availability of lifestyle programmes not always clear to neurologists	Navigators and/or clinical nurse practitioners can improve continuity and coordination of care
Role of neurologists in lifestyle management ‘unclear’	Proper on‐boarding with digital technology
	Shared care models with acute and primary care oversight of physiological monitoring during patient transition to community
	Embed co‐design in intervention and programme development

The inductive theme, ‘resourcing’, had most instances of simultaneous coding with the deductive themes, ‘barriers’ and ‘enablers’. Perceived barriers and enablers associated with resourcing were heterogeneous, relating to workforce, capacity, access and sustainability, across a range of contexts and models of care (Table 6).

**Table 6:** Simultaneous coding to themes: ‘Resourcing’, ‘barriers’ and ‘enablers’.

Barriers	Enablers
Lack of funding for translation into policy or practice	Leveraging existing services, with workforces that are sustainably funded, to implement novel interventions or programmes
Capacity of existing multimodal or behaviour change programmes to take people with stroke	Entrepreneurial approach to research, e.g., business cases, collaboration with private sector
Access to multimodal or behaviour change programmes currently extremely limited—and worse in regional and remote areas	Developing implementatio,n frameworks including programme logics and resource requirements
Safety concerns in multimodal programmes that include exercise	Building cost‐recovery mechanisms into interventions—e.g., utilising Federal funding structures such as MBS* item numbers
Staffing for multimodal or behaviour change programmes	Including economic evaluations in secondary prevention RCTs
Neurologists can generally only provide a single outpatient session to a person after their stroke, dedicating approximately 20 min to secondary prevention.	Models of care that include menus of options for delivery, including face‐to‐face, telehealth, eHealth and mHealth
Workforce—relatively few vascular neurologists and difficult to increase pool due to lack of interest in stroke	Adapting the Country Heart Attack Prevention (CHAP) model of care—which shifts cardiac rehabilitation to primary care with sustainable MBS funding—for stroke secondary prevention
Funding structure of MBS item numbers—opportunity cost for GPs if not providing short consults for secondary prevention	Potential for eHealth including mHealth to provide more cost‐effective and scalable secondary prevention
Constraints on use of some MBS item numbers in metropolitan areas	Including policymakers in implementation planning and discussion
Workforce: Difficulty attracting and retaining practice nurses	If multimodal or behaviour change programmes could be implemented in primary care, some impacts of shortage of vascular neurologists could be mitigated
Workforce: GP morale issues impact efforts to expand secondary prevention in primary care	New interventions or care models that add value to GPs' existing practices
Cost can be a barrier to GP attendance—impacts secondary prevention in primary care	GPs and practice nurses financially incentivised to adopt new care models or interventions

Abbreviations: GP, general practitioner; MBS, Medicare Benefits Schedule; RCT, randomised controlled trial.

* MBS items are funded through the Federal Government's Medicare universal health care system.

The inductive theme, ‘research translation pipeline’, had most instances of simultaneous coding with the deductive themes, ‘barriers’ and ‘enablers’. Perceived barriers and enablers associated with the research translation pipeline were diverse, relating to strategic considerations, clinical practice guidelines and responsibility for research translation, across a range of contexts and models of care (Table 7).

**Table 7:** Simultaneous coding to themes: ‘Research translation pipeline’, ‘barriers’ and ‘enablers’.

Barriers	Enablers
Too many pilot studies without fully powered RCTs to inform scale‐up or implementation	Strong evidence that physical activity, supervised exercise and diet are effective for modifying stroke risk factors
Lack of understanding of the mechanisms of action of secondary prevention interventions to assist with implementation	Co‐design methodologies involving people with stroke, carers, and clinicians, to enhance implementation of interventions
Strength of evidence of multimodal behaviour change programmes for secondary stroke prevention	Process and economic evaluations as part of development of interventions.
	Developing programmes with implementation and sustainment in mind from the outset
Implementation of evidence‐based programmes into practice is complex and context‐specific	Adapting or leveraging programmes or models that have been successfully implemented in similar CVDs
Existing clinical practice guidelines make only a weak recommendation for multimodal behaviour change programmes	More specific clinical practice guidelines
	DOACs
	Antiplatelets
	Blood pressure targets
Implementing clinical practice guideline recommendations is a complex, context‐specific problem; evidence‐based strategies are lacking	Using evidence‐based strategies for implementation, including identifying local champions and barriers, introducing audit feedback cycles and evaluation
Lack of a clear funding model that allows evidence to be translated into routine care	Involving implementation scientists and frameworks
Lack of clarity on who is responsible for research translation—researchers, health services, clinicians	Ensuring the needs of end users are well understood
Tension between the development of interventions and research with health service delivery of programmes	Building support from interest‐holders and thinking about sustainability throughout the research process
	Researchers partnering with NGOs to bridge gap between research and implementation
	Researchers developing business plans that leverage existing funding structures or include cost‐recovery mechanisms
	Quality improvement, including audit and feedback, to include secondary prevention processes of care and outcomes

Abbreviations: CVD, cardiovascular disease; DOAC, direct oral anticoagulants; NGO, non‐government organisations; RCT, randomised controlled trial.

**Figure 3:**
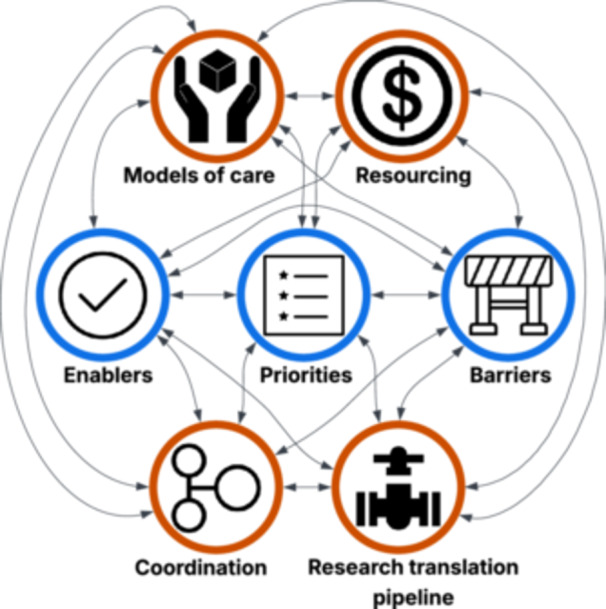
Connections between themes (blue = deductive, orange = inductive).

We identified 102 instances of simultaneous coding, which are summarised in Tables 4, 5, 6, 7. Eighty‐seven (85%) of these involved coding to three or more themes (see Supplement 2). For example, many summaries coded to ‘Resourcing’ and ‘Barriers’ were also coded to ‘Models of Care’ and/or to ‘Research Translation Pipeline’. All themes shared at least one such coded summary with every other theme (Figure 3).

## 4 Discussion

This roundtable aimed to use interest‐holder and lived experience discussions to generate a broad range of potential implementation and research priorities for stroke secondary prevention. As expected from our approach, which was not aimed at consensus building, the list of identified priorities was diverse and extensive, with no agreed shortlist. Summary points from the roundtable and small‐group discussions were coded to multiple themes, reflecting interrelationships between priorities, barriers, enablers, models of care, resourcing issues, issues relating to coordination and the research translation pipeline. These findings were made possible by our systems thinking approach, which was operationalised through the brief sent to participants and facilitated discussion of synergies, antagonisms and dependencies between priorities.

Broadly, the implementation and research priorities related to multimodal (e.g., supervised exercise plus education), cardiac rehabilitation and behaviour change programmes, care settings (including community, primary care and neurology) and leveraging digital modalities. Some of these priorities spanned aspects of care, including:

- Determining how programmes can be sustainably implemented

- Improving continuity of care across care transitions
- Increasing the reach and uptake of secondary prevention interventions through delivering personalised care.

There were some uncertainties relating to stroke secondary prevention programmes. Significantly, there was no straightforward answer to what should comprise the optimal programme for secondary prevention of stroke. The Stroke Foundation's Living Clinical Guidelines for Stroke Management make only a ‘weak recommendation’ that multimodal lifestyle programmes be used for secondary prevention [19]. The evidence that has informed this guideline recommendation is reinforced by a recent review of reviews, which identified that while there is moderate certainty evidence that multimodal programmes reduce cardiac events in people with stroke, there is limited evidence for their effects on recurrent stroke or overall mortality [25].

Other uncertainties included how to fund and resource stroke secondary prevention programmes, the health professions that should conduct them, and where such programmes should sit within the healthcare system. Contrasting perspectives were provided on how newer modalities (e.g., eHealth, mHealth)—and associated interventions—and different programmes (e.g., behaviour change or multimodal programmes) should be integrated within existing stroke services (e.g., neurology and traditional stroke rehabilitation) and primary care. There was ambivalence on whether a standardised programme for stroke secondary prevention was desirable, or whether it is preferable to be ‘platform‐agnostic’. In either case, participants agreed that there should be a national model of care for stroke secondary prevention, even if it was to be flexible, menu‐based and accommodate a range of platforms, interventions and smaller programmes.

Our findings on secondary prevention of stroke in Australia can be compared and contrasted to those of ACvA's Cardiac Rehabilitation and Secondary Prevention Roundtable, which identified five implementation priorities: standardised data collection for cardiac rehabilitation programmes; availability of flexible models for delivery of cardiac rehabilitation (including digital options) tailored to individual need and preference; utilisation of existing resources in primary care and hospital settings to create a menu of options for secondary prevention; better decision‐making for patient triage through algorithms; and the importance of system levers such as incentive payments. The focus on models that can provide personalised care and leverage existing services and system levers was shared with our roundtable. The ACvA's shortlist of implementation priorities was developed through a consensus‐building approach, which was well‐suited to the topic of Cardiac Rehabilitation, which is an established, standardised model of care. By contrast, we identified tensions and uncertainties, described above, that reflect the lack of an established model of care for secondary stroke prevention. This reality reconfirms the appropriateness of our systems thinking perspective and the aim of giving voice to a range of heterogeneous priorities and interrelationships between priorities.

Various perceived barriers to achieving secondary prevention priorities are related, in different ways, to coordination. A recurrent theme was the need for better coordination not only between clinicians in different parts of the health system, but between funders, researchers, policymakers, health services and people with stroke, their families and carers. A major system weakness identified was the lack of continuity between specialist stroke care and primary care, which has been identified in previous studies [26, 27]. One participant suggested that secondary prevention is ‘everyone's responsibility’ in the health system, but can thereby become ‘no‐one's responsibility’. Such ambiguity around accountability for preventing recurrent stroke was contrasted with secondary prevention for cardiac disease, which has a recognised national model of care—cardiac rehabilitation—that is funded and routinely implemented in subacute hospital settings throughout Australia [28]. Stroke secondary prevention does not have a standardised model of care. Participants suggested this is partly because stroke rehabilitation typically prioritises reducing impairment and disability through restoration or compensation techniques, rather than secondary prevention support and cardiovascular fitness [29]. Further, people with no impairment‐based goals, such as after TIA or mild stroke, can bypass rehabilitation altogether. This cohort remains at very high risk of recurrent stroke but can receive minimal secondary prevention support in the hospital system, as found in studies in other countries [26, 27]. These divergent clinical pathways result in responsibility for secondary prevention support falling, for different patients, to different levels of the health system. This likely contributes to disparities between people with stroke and people with cardiac disease, such as people with stroke being 37% more likely not to receive secondary prevention medications than people with coronary heart disease [12]. Similar disparities exist in other jurisdictions. In the United States, a 2006 study found that, compared to people after a cardiac event, people with a stroke were less likely to have used antithrombotic medication in the last year, ever been advised to exercise more, and ever been given appropriate dietary advice [30]. Cross‐sectional analyses of two large independent cohorts, one from the United Kingdom and one from the United States, found that people with stroke demonstrated poorer cardiovascular prevention profiles and lower adherence to therapies following clinical practice guidelines than patients with myocardial infarction [31]. We contend that, when it comes to CVD that has manifested in a cardiac event, the dual aims of *rehabilitation* and *secondary prevention* dovetail, making the single cardiac rehabilitation model suitable for fulfilling both aims. When it comes to CVD that has manifested as stroke, however, the dual aims of rehabilitation and secondary prevention do not necessarily neatly dovetail. Unfortunately, this reality is not reflected in Australian health strategy [32, 33]. This point is substantiated by our findings on barriers relating to coordination, which were made possible by our systems thinking focus and methodology.

Workforce, access, capacity and funding constraints were also perceived to hinder the implementation and sustainment of evidence‐based interventions and programmes to improve the secondary prevention of stroke. The health workforce in Australia is facing ongoing pressures, particularly in regional and rural Australia [33, 34, 35], where stroke incidence is 17% higher than in metropolitan areas [36]. To optimise secondary prevention of stroke, the roundtable identified that capacity needs to be built in vascular neurology, general practice, primary care nursing and allied health, including exercise physiology and dietetics. Digital health, including mHealth, can improve equity of access to secondary prevention support, including self‐management at scale, and should be leveraged to mitigate resourcing constraints related to the secondary prevention of stroke [37].

Perceived barriers relating to coordination and resourcing converged with barriers associated with the research translation pipeline. A tension emerged between prioritising implementation of behaviour change support (‘lifestyle’) interventions and the perceived need for further evidence of effectiveness before implementation occurs. This raises the question of the type of research studies that could be done to provide evidence of effectiveness and be embedded within the health system, as we elaborate below. Federal health policies on heart disease and stroke [32], prevention [33] and primary care [34] identify as priorities the translation of evidence into clinical practice and/or the undertaking of translational research. However, roundtable participants emphasised the lack of a clear funding model for translating evidence into routine care. Research Translation Centres have been established in Australia over the last decade, with accreditation provided by the National Health and Medical Research Council [38]. These centres have an explicit remit of facilitating research translation and building capacity in implementation research, knowledge mobilisation and brokering, evaluation and collaborative priority‐setting [39]. Nonetheless, in our roundtable, opinions diverged on who is responsible for research translation, particularly the role of the researcher. A feasible suggestion—that could have a high impact downstream—was that programmes should be developed by researchers with implementation and sustainment in mind from the outset, although such development itself requires adequate funding. Recent national and international discussions have identified the need to strengthen learning health systems (LHSs) in stroke. LHSs could address research translation and implementation gaps by establishing, at a system level, healthcare that incorporates patient perspectives, effectively gathers and utilises data, produces knowledge, translates evidence into practice, and continuously improves service quality [40]. A recent topical review suggested that many stroke services may deploy LHS concepts operationally, but without using an explicit LHS framework or communicating their knowledge through academic publication [40]. The elements already exist within Australia, then, for an integrated, systems‐based approach to closing evidence‐practice gaps [41].

We conclude that secondary prevention of stroke in Australia is not a merely ‘complicated’ problem that could be effectively dealt with by addressing various contributing factors independently [42]. Rather, stroke secondary prevention is a ‘wicked problem’ involving a complex system, comprised of interacting funding, policy, primary care, secondary care, community, governance, workforce, communication, technological and personal elements. Other wicked problems that have been identified include Alzheimer's disease in the United States [43], CVD prevention [44] and diabetes prevention [45] in Finland, obesity prevention in the Netherlands [46], and the combination of multimorbidity, inequity and fragmented health services in developed nations [47]. These problems have been ameliorated through multilevel interventions aimed at effecting system‐level change, including dietary reform, tobacco control, community mobilisation, policy reform, media campaigns, the creation of incentives and disincentives (such as taxes), data collection and evaluation, and the coordination of health and social services through Integrated Care frameworks [43, 44, 45, 46, 47].

Australian Government strategies on stroke and heart disease [32], primary care [34] and preventive health [33] state that systems‐based approaches are needed, without specifying how this should occur. Consistent with previous approaches that have successfully ameliorated wicked problems, greater adoption of an LHS approach, including embedding real‐world monitoring of stroke secondary prevention, could help operationalise a systems approach. To overcome barriers related to fragmentation of the health system, including inadequate communication and coordination of care, a model of care for stroke secondary prevention with a systems focus is needed. Whether the model is centralised or decentralised, standardised or ‘platform‐agnostic’, it must account for these complexities and clearly define responsibilities for stroke secondary prevention across the range of stroke presentations and pathways. A model of care could help reduce perceived inequities that exist for people with stroke, compared to people with cardiac disease, in accessing dedicated secondary prevention programmes, and in terms of equitable sharing of responsibility across various levels of the health system.

In the shorter term, roundtable participants described various enablers of stroke secondary prevention priorities that could act at system leverage points. These include:

- Leveraging digital health and existing services, infrastructure and funding structures to develop innovative programmes and care models.
- Centralising effective services while planning for adaptation to local contexts.
- Developing programmes or models of care that consider how responsibility for stroke secondary prevention is distributed across different levels of the health system.
- Providing personalised care by offering flexible menus of options.
- Developing sound business models for programmes.
- Vertical and horizontal partnering, including across sectors and levels of the health system.
- Dedicated funding for implementation, scale‐up and sustainment of effective programmes and services.
- Planning for implementation and sustainment during initial programme development.
- Co‐design methodologies that engage all relevant interest‐holders.
- More specific clinical practice guideline recommendations to close the gap between evidence and practice.

Following the roundtable, various participants have drawn on these enablers in practice. Several have partnered with each other and with the Stroke Foundation to develop innovative programmes that leverage the enablers listed above, to target identified problems in stroke secondary prevention and aim at sustained real‐world implementation. In these ways, progress is being made towards solving the ‘wicked problem’ of stroke secondary prevention. Having canvassed a heterogenous range of views in this roundtable in order to capture the complexity of stroke secondary prevention, next a roundtable is needed that is informed by systems thinking but aimed at establishing consensus for a model of care for stroke secondary prevention in Australia.

## Author Contributions

**Seamus Barker:** conceptualisation, investigation, data curation, analysis, methodology, project administration, writing – original draft preparation, review and editing. **Dominique Cadilhac:** conceptualisation, investigation, methodology, validation, writing – review and editing. **Jan Cameron:** writing – review and editing. **Marie‐Louise Bird:** investigation, writing – review and editing. **Liam Johnson:** investigation, writing – review and editing. **Coralie English:** investigation, writing – review and editing. **Kyra Hamilton:** investigation, writing – review and editing. **Robyn A. Clark:** investigation, writing – review and editing. **Timothy Kleinig:** investigation, writing – review and editing. **Stephanie Smith:** investigation, validation, writing – review and editing. **Beth Crane:** validation, writing – review and editing. **Brian Beh:** investigation. **Kim Beesley:** investigation, validation. **Jennifer Muller:** investigation, validation. **Ramesh Sahathevan:** investigation, writing – review and editing. **Helen Dewey:** investigation. **Carlos Garcia‐Esperon:** investigation. **Mark Nelson:** investigation, writing – review and editing. **Seana Gall:** conceptualisation, funding acquisition, investigation, methodology, supervision, validation, writing – original draft preparation, review and editing.

## Ethics Statement

This study was approved by the University of Tasmania Human Research Ethics Committee, and all participants provided written, informed consent (H0028678).

## Conflicts of Interest

The authors declare no conflicts of interest.

## Supporting information

Supporting File

## Data Availability

The data that support the findings of this study are available on request from the corresponding author. The data are not publicly available due to their containing information that could compromise the privacy of research participants.
