# Supplementary material for: “Everyone's Responsibility and No‐One's Responsibility”: A Thematic Analysis of a Roundtable on the Complex Problem of Secondary Stroke Prevention in Australia
Source: Health Expect. 2026 Jul 6;29(4):e70755. doi: 10.1111/hex.70755 (PMC13338573; doi:10.1111/hex.70755)
Supplement: Supplementary file 1 — Supporting File [file HEX-29-e70755-s001.docx]

**Supplementary Material**

Supplement I: Roundtable Participant Brief

Supplement II: Consolidated criteria for reporting qualitative studies (COREQ)

Supplement III: Summaries of discussion points coded to multiple themes

**Supplement 1: Stroke Secondary Prevention Roundtable—Participant Brief**

**SSPR Purpose:** The purpose of the SSPR is to bring together a diverse range of stakeholders with an interest in stroke secondary prevention, including researchers, clinicians, people with lived experience of stroke, and representatives from the Australian Stroke Foundation, Australian Cardiovascular Alliance, and State and Federal Government Departments of Health.

Eight broad topics in stroke secondary prevention have been identified by the STOPstroke team (see below). Certain participants (indicated below) are invited to provide individual presentations, of up to ten minutes in length, on the basis that their experience and expertise aligns with one of these eight topics (without necessarily being limited to that topic).

In these individual presentations, participants are encouraged to outline what they see as priorities for implementation and/or research in stroke secondary prevention, in relation to their allocated topic. In so doing, participants may wish to refer to their own work, in some cases quite specific projects (see below).

In the second half of the SSPR, moderated discussion will take place, which will help identify and elaborate points of connection between different priorities and issues, including as these traverse health policy, primary care, secondary care, research, and end-user/lived experience. Open discussion will be encouraged, although some themes, or topics, will be introduced to help facilitate conversation (see below). Participants are also welcome to introduce topics that were not addressed in individual presentations, such as relate to their own areas of expertise.

The SSPR will be audio-recorded. Following the SSPR, the audio-recording will be professionally transcribed, in a de-identified form. A thematic analysis will be conducted by the research team, to extract and analyse the themes and sub-themes that emerged from the discussions. The results will be published in an appropriate academic journal, and may be presented at academic conferences and/or in public fora. A Participant Information Sheet and Consent Form will be circulated shortly.

**Topics for individual presentations on stroke secondary prevention priorities:**

**Topic 1**: Community—Marie-Louise Bird PhD

**Topic 2**: Health systems—Liam Johnson PhD

**Topic 3**: mHealth—Prof Dominique Cadilhac

**Topic 4**: eHealth—iRebound: Prof Coralie English

**Topic 5**: Behaviour change—Living Well After Stroke: Stephanie Smith PhD, A/Prof Kyra Hamilton, A/Prof Joy Parkinson

**Topic 6**: Primary care – Country Heart Attack Prevention (CHAP): Prof Robyn Clark

**Topic 7**: Continuity of care – Stroke Navigator: Luke Hays

**Topic 8:** Health policy (REDACTED)

**Key questions to consider, in preparing your *individual presentation* (up to ten minutes in length):**

- What do you consider to be the priorities for implementation and/or research, for stroke secondary prevention?
- Are these priorities currently being implemented and/or studied (for example, in your own work)?
- What barriers exist to the achievement of these implementation and/or research priorities?
- What factors would enable the achievement of these implementation and/or research priorities?

**Desired outcomes of individual presentations**

- The group becomes aware of a diversity of views on priorities for stroke secondary prevention implementation and/or research, coming from researchers, clinicians, people with lived experience, and representatives of Government and peak bodies.

**Moderated discussion:**

The second half of the SSPR will involve moderated discussion. The purpose is not so much to rank priorities for stroke secondary prevention, but to break out of traditional silos and develop understanding of how these priorities are connected. All participants are invited to contribute to the discussion.

**Key questions to consider, in preparation for the SSPR moderated discussion**:

- What needs to happen for evidence-based interventions for stroke secondary prevention to be implemented and brought to scale?
- What connections (synergies, antagonisms, dependencies) might exist between different stroke secondary prevention priorities, coming from policy, research, implementation, primary care, secondary care, and lived experience?
- What would a “blue sky” (no limits) solution look like, to achieve your identified priorities for stroke secondary prevention?
- What concrete, realistic changes could be made – whether in policy, funding, health system organisation, or anything else – that would help enable the achievement of your identified priorities?

**Desired outcomes of moderated discussion**:

- Through a collaborative process, identify and elaborate points of connection between the priorities and issues that have been raised, including as these traverse health policy, primary care, secondary care, research, and end user/lived experience.
- Through a collaborative process, develop a preliminary sense of what might be required for implementation and research priorities, in stroke secondary prevention, to be achieved.

**Next steps:**

Following the SSPR, the audio-recording of the event will be transcribed, in a de-identified form. A thematic analysis will then be undertaken by the STOPstroke team, and these results developed for publication.

**Background**

*STOPstroke and secondary prevention*

The proposed SSPR sits within the STOPstroke Synergy Grant. STOPstroke is a five-year NHMRC-funded Synergy Grant (CIA A/Prof Seana Gall, University of Tasmania), which aims to identify those at risk of stroke; to improve the management of those at risk of stroke; and develop real-world modelling to understand the effectiveness of stroke prevention.

Stroke is the second most common cause of death and third leading cause of disability in the world. Stroke is also a highly preventable disease affecting all ages, ethnicities, and socio-economic groups. Due to ongoing ageing of the population, population growth and a trend towards increasing prevalence of many important risk factors for stroke, the burden of stroke is likely to continue increasing, unless cost-effective primary stroke preventative strategies can be found and implemented.

Secondary prevention of stroke can be enhanced through the modification of known risk factors, but many questions remain unanswered regarding how strategies aimed at risk factor modification can best be implemented.

*STOPstroke’s Preventing Stroke Research Priorities Project*

The STOPstroke team based at the Menzies Institute for Medical Research, University of Tasmania, in 2022 undertook a Preventing Stroke Research Priorities Project. This project sought the opinions of stroke survivors, carers, and clinicians regarding their priorities for research, in aid of stroke prevention. The project used the James Lind Alliance methodology for Priority Setting Partnerships. Through this methodology, the purpose of this project was to achieve consensus regarding a list of priorities for research in stroke prevention.

The STOPstroke Stroke Secondary Prevention Roundtable builds upon this earlier work, but has a different purpose and methodology, incorporating a focus on implementation, and using roundtable discussion to elaborate some of the barriers to, and enablers of, the achievement of secondary prevention priorities, and points of connection between different priorities that traverse different sectors and levels of healthcare, rather than creating consensus around a list of research priorities.

*Australian Cardiovascular Alliance Roundtables*

The Australian Cardiovascular Alliance has previously conducted roundtables, including a Joint National Cardiovascular Implementation and Policy Roundtable, and a Cardiac Rehabilitation and Secondary Prevention Roundtable: Australian Implementation and Research Priorities.

The STOPstroke SSPR seeks to build upon the valuable work already completed by the Australian Cardiovascular Alliance, and we acknowledge the influence of their formats upon our own event, including the focus of their Cardiac Rehabilitation Roundtable on priorities for implementation and research. In restricting the scope of the STOPstroke SSPR to stroke secondary prevention, and focusing the moderated discussion on points of connection (synergies, antagonisms, dependencies) between different priorities, we aim to fill a gap in the understanding of stroke secondary prevention, and make a novel contribution to the literature.

**Supplement 2:** **Consolidated criteria for reporting qualitative studies (COREQ): 32-item checklist**

| **No. Item** | **Guide questions/description** | **Reported on Page #** |
| --- | --- | --- |
| **Domain 1: Research team and reﬂexivity** |  |  |
| *Personal Characteristics* |  |  |
| 1. Inter viewer/facilitator | Which author/s conducted the inter view or focus group? | 14 |
| 2. Credentials | What were the researcher’s credentials? E.g. PhD, MD | 14 |
| 3. Occupation | What was their occupation at the time of the study? | 14 |
| 4. Gender | Was the researcher male or female? | 14 |
| 5. Experience and training | What experience or training did the researcher have? | 14 |
| *Relationship with participants* |  |  |
| 6. Relationship established | Was a relationship established prior to study commencement? | 10 |
| 7. Participant knowledge of the interviewer | What did the participants know about the researcher? e.g. personal goals, reasons for doing the research | 14 |
| 8. Interviewer characteristics | What characteristics were reported about the inter viewer/facilitator? e.g. Bias, assumptions, reasons and interests in the research topic | 14 |

| **Domain 2: study design** |  |  |
| --- | --- | --- |
| *Theoretical framework* |  |  |
| 9. Methodological orientation and Theory | What methodological orientation was stated to underpin the study? e.g. grounded theory, discourse analysis, ethnography, phenomenology, content analysis | 9, 13 |
| *Participant selection* |  |  |
| 10. Sampling | How were participants selected? e.g. purposive, convenience, consecutive, snowball | 10 |
| 11. Method of approach | How were participants approached? e.g. face-to-face, telephone, mail, email | 10 |
| 12. Sample size | How many participants were in the study? | 14 |
| 13. Non-participation | How many people refused to participate or dropped out? Reasons? | 11 |
| *Setting* |  |  |
| 14. Setting of data collection | Where was the data collected? e.g. home, clinic, workplace | 14 |
| 15. Presence of non-participants | Was anyone else present besides the participants and researchers? | 14 |
| 16. Description of sample | What are the important characteristics of the sample? e.g. demographic data, date | 14-15 |
| *Data collection* |  |  |
| 17. Interview guide | Were questions, prompts, guides provided by the authors? Was it pilot tested? | 12 |
| 18. Repeat interviews | Were repeat interviews carried out? If yes, how many? | 11,12,16 |
| 19. Audio/visual recording | Did the research use audio or visual recording to collect the data? | 13 |
| 20. Field notes | Were ﬁeld notes made during and/or after the interview or focus group? | 13 |
| 21. Duration | What was the duration of the interviews or focus group? | 15 |
| 22. Data saturation | Was data saturation discussed? | 14 |
| 23. Transcripts returned | Were transcripts returned to participants for comment and/or correction? | 14 |
| **Domain 3: analysis and ﬁndings** |  |  |
| *Data analysis* |  |  |
| 24. Number of data coders | How many data coders coded the data? | 13 |
| 25. Description of the coding tree | Did authors provide a description of the coding tree? | 13, Supplement 2 |
| 26. Derivation of themes | Were themes identiﬁed in advance or derived from the data? | 13 |
| 27. Software | What software, if applicable, was used to manage the data? | 13 |
| 28. Participant checking | Did participants provide feedback on the ﬁndings? | 14 |
| *Reporting* |  |  |
| 29. Quotations presented | Were participant quotations presented to illustrate the themes/ﬁndings? Was each quotation identiﬁed? e.g. participant number | No but justification for why provided p 19. |
| 30. Data and ﬁndings consistent | Was there consistency between the data presented and the ﬁndings? | Yes, 26-34 |
| 31. Clarity of major themes | Were major themes clearly presented in the ﬁndings? | Yes 17-19 |
| 32. Clarity of minor themes | Is there a description of diverse cases or discussion of minor themes? | Tensions in findings (diverse cases) discussed extensively 26-34 |

**Supplement 3: Summaries of discussion points coded to multiple themes** (✓ = coded to that theme)

|  | **Themes** | | | | | | | |
| --- | --- | --- | --- | --- | --- | --- | --- | --- |
| **Discussion points**  **summaries** | **Models of care** | **Research priority** | **Implementation priority** | **Coordination** | **Resourcing** | **Research translation pipeline** | **Barriers** | **Enablers** |
| Determining the most effective and cost-effective program model (e.g. supervised exercise plus education) | ✓ | ✓ |  |  | ✓ | ✓ |  |  |
| Determining the “active ingredients” of successful multimodal interventions | ✓ | ✓ |  |  |  |  |  |  |
| Access to multimodal programs whenever the person living with stroke is ready—such programs being available at different points in care continuum | ✓ |  | ✓ | ✓ | ✓ |  |  |  |
| Determining whether a ‘cardiac rehabilitation’ model is appropriate for stroke secondary prevention | ✓ | ✓ |  |  |  |  |  |  |
| Equity for people with stroke having access to state-funded secondary prevention comparable to cardiac rehabilitation for cardiac patients | ✓ |  | ✓ |  | ✓ |  |  |  |
| New service models to support behaviour change | ✓ |  | ✓ |  |  |  |  |  |
| Evaluate programs that develop behaviour change capacities | ✓ | ✓ |  |  |  |  |  |  |
| Translate evidence-based behaviour change programs into usual care | ✓ |  | ✓ |  |  | ✓ |  |  |
| Personalised, culturally safe lifestyle medicine | ✓ |  | ✓ | ✓ | ✓ |  |  |  |
| Producing evidence into effectiveness and cost-effectiveness of different mHealth interventions compared with usual care | ✓ | ✓ |  |  | ✓ | ✓ |  |  |
| As high level evidence is gathered, integrating mHealth interventions synergistically with other service models as part of usual care | ✓ |  | ✓ | ✓ |  | ✓ |  |  |
| Pipeline from pilot studies to fully powered trials to translation into routine care | ✓ |  | ✓ |  |  | ✓ |  |  |
| eHealth resources for behaviour change support should focus on implementation rather than gathering evidence of effectiveness | ✓ |  | ✓ |  |  | ✓ |  |  |
| Integrating eHealth with other interventions in larger rehabilitation and community service models | ✓ |  | ✓ | ✓ |  |  |  |  |
| After stroke, every patient should have a timely follow-up with a neurologist with expertise in stroke for a secondary prevention plan | ✓ |  | ✓ | ✓ | ✓ | ✓ |  |  |
| National, multidisciplinary, evidence-based, multimodal behaviour change program for people with stroke | ✓ |  | ✓ | ✓ |  | ✓ |  |  |
| Personalised secondary prevention targets | ✓ |  | ✓ | ✓ |  |  |  |  |
| Clinical practice guidelines that have clear, evidence-based recommendations for direct oral anticoagulants (DOACs), antiplatelets and blood pressure, glucose and lipid targets | ✓ |  | ✓ |  |  | ✓ |  |  |
| Ensuring neurologists remain up to date with evidence-based treatment options, including for glucose and lipid management | ✓ |  | ✓ |  |  | ✓ |  |  |
| GPs involved in behaviour change support and overseeing secondary prevention management in the community | ✓ |  | ✓ | ✓ |  |  |  |  |
| Determining how programs can be sustainably implemented | ✓ | ✓ |  | ✓ | ✓ | ✓ |  |  |
| Improving reach and uptake of secondary prevention interventions and programs through personalised care | ✓ |  | ✓ | ✓ |  | ✓ |  |  |
| Building evidence vs translation or implementation | ✓ | ✓ | ✓ | ✓ |  | ✓ |  |  |
| Single national program for stroke secondary prevention vs plurality of interventions and programs | ✓ |  | ✓ | ✓ |  | ✓ |  |  |
| The role of the researcher in implementation and translation | ✓ |  |  | ✓ |  | ✓ |  |  |
| Establishing evidence for novel primary care models for stroke secondary prevention | ✓ | ✓ |  | ✓ |  | ✓ |  |  |
| Fragmentation of health system | ✓ |  |  | ✓ |  |  | ✓ |  |
| Minimal coordination between specialists; specialists and GPs; GPs and allied health; researchers and policymakers; researchers and funders | ✓ |  |  | ✓ | ✓ | ✓ | ✓ |  |
| No clear pathway for implementation of research into routine care |  |  |  | ✓ | ✓ | ✓ | ✓ |  |
| Inadequate communication between levels and services of health system; clinicians, families and caregivers | ✓ |  |  | ✓ |  |  | ✓ |  |
| Different clinical pathways after stroke/TIA for those going to rehabilitation or directly home creates complexity in coordination across levels and specialities within health care system | ✓ |  |  | ✓ |  |  | ✓ |  |
| No standardised model of care for stroke secondary prevention results in lack of coordinated care across hospital and primary care, in contrast with post cardiac event. | ✓ |  |  | ✓ |  | ✓ | ✓ |  |
| Responsibility of many disciplines and parts of the health and community sectors | ✓ |  |  | ✓ |  |  | ✓ |  |
| Despite common risk factors for different chronic conditions, particularly cardiac conditions, health system divides by disease and body system | ✓ |  |  | ✓ |  |  | ✓ |  |
| Behaviour change is difficult to achieve |  |  |  | ✓ |  |  | ✓ |  |
| Low technology literacy in patients |  |  |  | ✓ |  |  | ✓ |  |
| Patient preference |  |  |  | ✓ |  |  | ✓ |  |
| Need for oversight of physiological monitoring through mHealth and eHealth | ✓ |  |  | ✓ | ✓ |  | ✓ |  |
| Availability of lifestyle programs not always clear to neurologists | ✓ |  |  | ✓ |  |  | ✓ |  |
| Role of neurologists in lifestyle management “unclear” | ✓ |  |  | ✓ |  |  | ✓ |  |
| Embedding patient autonomy and choice (person-centred care) within stroke secondary prevention programs to improve partnering between patients and clinicians | ✓ |  |  | ✓ |  |  |  | ✓ |
| “Shared care” models to improve partnering between different treating clinicians | ✓ |  |  | ✓ |  |  |  | ✓ |
| Communication between specialists and general practice can be improved through standardisation and automation of letters |  |  |  | ✓ |  |  |  | ✓ |
| Creating more partnerships between stakeholders, clinicians, patients and families, including through formal multi-disciplinary teams | ✓ |  |  | ✓ |  |  |  | ✓ |
| Cardiac rehabilitation model of care featuring menu of options can enhance reach and uptake—could be adapted for stroke | ✓ |  |  | ✓ |  |  |  | ✓ |
| Cardiac rehabilitation model set in primary care can enhance reach and uptake—could be adapted for stroke | ✓ |  |  | ✓ |  |  |  | ✓ |
| Model of care using existing Medicare Benefits Schedule (MBS) item numbers to coordinate multidisciplinary, multimodal primary care (like primary care-based cardiac rehabilitation model) | ✓ |  |  | ✓ | ✓ |  |  | ✓ |
| Programs that develop behaviour change capabilities | ✓ |  |  | ✓ |  |  |  | ✓ |
| Peer-led models for secondary prevention support | ✓ |  |  | ✓ |  |  |  | ✓ |
| Family and community members supporting transition to community living | ✓ |  |  | ✓ |  |  |  | ✓ |
| Clinician training to form effective partnerships with families and carers | ✓ |  |  | ✓ |  |  |  | ✓ |
| Clinical practice guidelines requiring clinicians to form effective partnerships with families and carers | ✓ |  |  | ✓ |  | ✓ |  | ✓ |
| Navigators and/or clinical nurse practitioners can improve continuity and coordination of care | ✓ |  |  | ✓ |  |  |  | ✓ |
| Proper on-boarding with digital technology |  |  |  | ✓ |  |  |  | ✓ |
| Shared care models with acute and primary care oversight of physiological monitoring during patient transition to community | ✓ |  |  | ✓ |  |  |  | ✓ |
| Embed co-design in intervention and program development |  |  |  | ✓ |  | ✓ |  | ✓ |
| Lack of funding for translation into policy or practice | ✓ |  |  | ✓ | ✓ | ✓ | ✓ |  |
| Capacity of existing multimodal or behaviour change programs to take people with stroke | ✓ |  |  |  | ✓ |  | ✓ |  |
| Access to multimodal or behaviour change programs currently extremely limited – and worse in regional and remote areas | ✓ |  |  |  | ✓ |  | ✓ |  |
| Safety concerns in multimodal programs that include exercise | ✓ |  |  |  | ✓ |  | ✓ |  |
| Staffing for multimodal or behaviour change programs | ✓ |  |  |  | ✓ |  | ✓ |  |
| Neurologists can generally only provide a single outpatient session to a person after their stroke, dedicating approximately 20 minutes to secondary prevention. | ✓ |  |  |  | ✓ |  | ✓ |  |
| Workforce – relatively few vascular neurologists and difficult to increase pool due to lack of interest in stroke |  |  |  |  | ✓ |  | ✓ |  |
| Funding structure of MBS item numbers – opportunity cost for GPs if not providing short consults for secondary prevention | ✓ |  |  |  | ✓ |  | ✓ |  |
| Constraints on use of some MBS item numbers in metropolitan areas | ✓ |  |  |  | ✓ |  | ✓ |  |
| Workforce: difficulty attracting and retaining practice nurses |  |  |  |  | ✓ |  | ✓ |  |
| Workforce: GP morale issues impact efforts to expand secondary prevention in primary care | ✓ |  |  |  | ✓ |  | ✓ |  |
| Cost a barrier to GP attendance – impacts secondary prevention in primary care | ✓ |  |  |  | ✓ |  | ✓ |  |
| Leveraging existing services, with workforces that are sustainably funded, to implement novel interventions or programs | ✓ |  |  | ✓ | ✓ | ✓ |  | ✓ |
| Entrepreneurial approach to research, e.g. business cases, collaboration with private sector | ✓ |  |  | ✓ | ✓ |  |  | ✓ |
| Developing implementation frameworks including program logics and resource requirements |  |  |  |  | ✓ | ✓ |  | ✓ |
| Building cost-recovery mechanisms into interventions—e.g. utilising Federal funding structures such as Medicare Benefits Schedule (MBS)* item numbers | ✓ |  |  |  | ✓ |  |  | ✓ |
| Including economic evaluations in secondary prevention (Randomised Controlled Trials (RCTs) |  |  |  |  | ✓ | ✓ |  | ✓ |
| Models of care that include menus of options for delivery, including face-to-face, telehealth, eHealth, and mHealth | ✓ |  |  |  | ✓ |  |  | ✓ |
| Adapting the Country Heart Attack Prevention (CHAP) model of care—which shifts cardiac rehabilitation to primary care with sustainable MBS funding—for stroke secondary prevention | ✓ |  |  | ✓ | ✓ | ✓ |  | ✓ |
| Potential for eHealth including mHealth to provide more cost-effective and scalable secondary prevention | ✓ |  |  |  | ✓ | ✓ |  | ✓ |
| Including policymakers in implementation planning and discussion |  |  |  | ✓ | ✓ | ✓ |  | ✓ |
| If multimodal or behaviour change programs could be implemented in primary care, some impacts of shortage of vascular neurologists could be mitigated | ✓ |  |  |  | ✓ |  |  | ✓ |
| New interventions or care models that add value to GPs’ existing practices | ✓ |  |  |  | ✓ |  |  | ✓ |
| GPs and practice nurses financially incentivised to adopt new care models or interventions | ✓ |  |  |  | ✓ |  |  | ✓ |
| Too many pilot studies without fully powered RCTs to inform scale up or implementation |  |  |  |  |  | ✓ | ✓ |  |
| Lack of understanding of the mechanisms of actions of secondary prevention interventions to assist with implementation | ✓ |  |  |  |  | ✓ | ✓ |  |
| Strength of evidence of multimodal behaviour change programs for secondary stroke prevention | ✓ |  |  |  |  | ✓ | ✓ |  |
| Implementation of evidence-based programs into practice is complex and context specific | ✓ |  |  | ✓ |  | ✓ | ✓ |  |
| Existing clinical practice guidelines make only a weak recommendation for multimodal behaviour change programs | ✓ |  |  |  |  | ✓ | ✓ |  |
| Lack of clear funding model that allows evidence to be translated into routine care | ✓ |  |  | ✓ | ✓ | ✓ | ✓ |  |
| Lack of clarity on who is responsible for research translation—researchers, health services, clinicians |  |  |  | ✓ |  | ✓ | ✓ |  |
| Tension between development of interventions and research with health service delivery of programs |  |  |  | ✓ |  | ✓ | ✓ |  |
| Strong evidence that physical activity, supervised exercise and diet are effective for modifying stroke risk factors | ✓ |  |  |  |  | ✓ |  | ✓ |
| Co-design methodologies involving people with stroke, carers, and clinicians, to enhance implementation of interventions |  |  |  | ✓ |  | ✓ |  | ✓ |
| Process and economic evaluations as part of development of interventions |  |  |  |  |  | ✓ |  | ✓ |
| Developing programs with implementation and sustainment in mind from the outset | ✓ |  |  | ✓ | ✓ | ✓ |  | ✓ |
| Adapting or leveraging programs or models that have been successfully implemented in similar CVDs | ✓ |  |  |  | ✓ | ✓ |  | ✓ |
| More specific clinical practice guidelines   - DOACs - Antiplatelets - Blood pressure targets | ✓ |  |  |  |  | ✓ |  | ✓ |
| Using evidence-based strategies for implementation including identifying local champions and barriers, introducing audit feedback cycles and evaluation |  |  |  |  |  | ✓ |  | ✓ |
| Involving implementation scientists and frameworks |  |  |  |  |  | ✓ |  | ✓ |
| Ensuring the needs of end users are well understood |  |  |  | ✓ |  | ✓ |  | ✓ |
| Building in support from stakeholders and thinking about sustainability throughout the research process |  |  |  | ✓ |  | ✓ |  | ✓ |
| Researchers partnering with NGOs to bridge gap between research and implementation |  |  |  | ✓ |  | ✓ |  | ✓ |
| Researchers developing business plans that leverage existing funding structures or include cost-recovery mechanisms | ✓ |  |  | ✓ | ✓ | ✓ |  | ✓ |
| Quality improvement including audit and feedback to include secondary prevention processes of care and outcomes |  |  |  |  |  | ✓ |  | ✓ |
